# Circulating microRNAs and Bioinformatics Tools to Discover Novel Diagnostic Biomarkers of Pediatric Diseases

**DOI:** 10.3390/genes8090234

**Published:** 2017-09-19

**Authors:** Antonella Baldassarre, Cristina Felli, Giorgio Prantera, Andrea Masotti

**Affiliations:** 1 Bambino Gesù Children’s Hospital-IRCCS, Research Laboratories, 00146 Rome, Italy; antonella.baldassarre@opbg.net (A.B.); cristina.felli@opbg.net (C.F.); 2 Department of Ecology and Biology, Università della Tuscia, 01100 Viterbo, Italy; prantera@unitus.it

**Keywords:** circulating microRNAs, biomarkers, bioinformatics tools, pediatric diseases, body fluids

## Abstract

MicroRNAs (miRNAs) are small noncoding RNAs that regulate gene expression at the post-transcriptional level. Current studies have shown that miRNAs are also present in extracellular spaces, packaged into various membrane-bound vesicles, or associated with RNA-binding proteins. Circulating miRNAs are highly stable and can act as intercellular messengers to affect many physiological processes. MicroRNAs circulating in body fluids have generated strong interest in their potential use as clinical biomarkers. In fact, their remarkable stability and the relative ease of detection make circulating miRNAs ideal tools for rapid and non-invasive diagnosis. This review summarizes recent insights about the origin, functions and diagnostic potential of extracellular miRNAs by especially focusing on pediatric diseases in order to explore the feasibility of alternative sampling sources for the development of non-invasive pediatric diagnostics. We will also discuss specific bioinformatics tools and databases for circulating miRNAs focused on the identification and discovery of novel diagnostic biomarkers of pediatric diseases.

## 1. Biogenesis and Regulatory Functions of microRNAs

MicroRNAs (miRNAs) are short non-coding RNAs that are widely recognized as fundamental regulators of several biological and cellular functions [1,2]. In the early 1990s, miRNAs were first discovered in *Caenorhabditis elegans* as small RNA molecules that regulate the developmental fates of these organisms [3]. Since then, many miRNAs were discovered and ascertained to regulate not only the developmental stages, but also many other cellular functions, such as apoptosis, proliferation and differentiation. To date, 1881 precursors and 2588 mature miRNAs (miRBase release 21, June 2014) have been annotated in humans.

Long primary miRNA transcripts (pri-miRNA) are initially transcribed by RNA polymerase II in the nucleus, then cleaved to precursor hairpin (pre-miRNA) by the microprocessor complex Drosha and DGCR8. Pre-miRNAs are exported into the cytoplasm (by Exportin 5 and other cofactors) and further cleaved to mature miRNAs by the RNase III endonuclease Dicer. One of the two RNA strands of the mature miRNA is incorporated into the RNA-induced silencing complex (RISC) and guided to bind the 3’ untranslated regions of the “target” mRNA. After recognition, this interaction leads to a translational repression or mRNA degradation and, as a consequence, protein and gene expression are reduced or abolished [4]. This complex system has been demonstrated to be a fine regulatory machinery, conserved in many species and evolutionarily designed to control gene expression efficiently.

## 2. Biogenesis, Stability and Cellular Recognition of Circulating microRNAs

**Figure 1:**
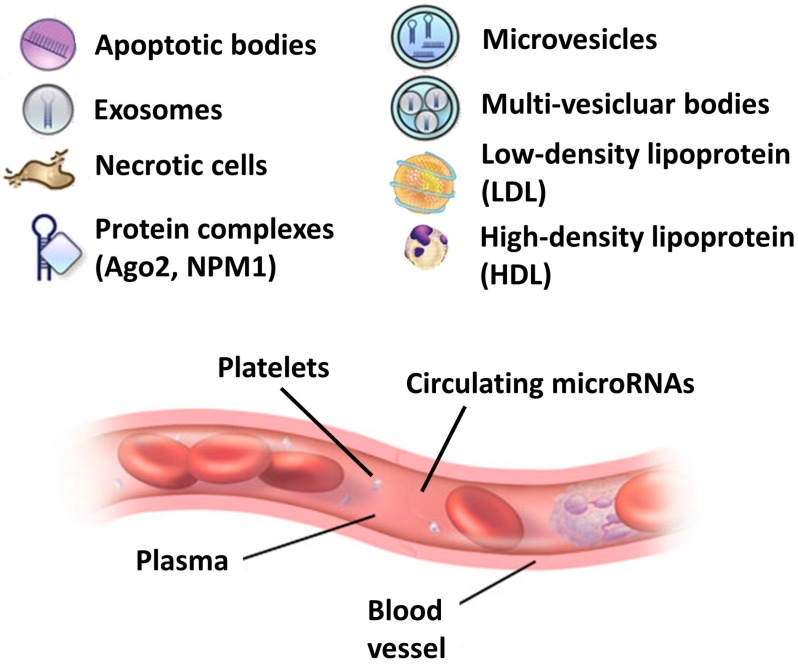
Different species of circulating MicroRNAs (miRNAs) in the bloodstream.

In the last few years, the discovery of miRNAs in biological fluids has generated a great interest in their potential use as biomarkers. Circulating biomarkers play a significant role in clinical applications, especially for the diagnosis of specific diseases, to monitor the therapeutic effect of a drug, or to predict tumor recurrence in chemotherapy-treated patients [5]. As an example, Chim and coworkers were the first to identify circulating placental miRNAs in the plasma of pregnant women [6]. Circulating miRNAs have many of the essential characteristics of good biomarkers: they are stable in the circulation and resistant to RNAses digestion, extreme pH, high temperatures, extended storage, and multiple freeze–thaw cycles [7,8]. In many cases, changes in circulating miRNA expression levels have been associated with different diseases or certain biological/pathological stages. Circulating miRNAs are released into the bloodstream in many forms (Figure 1), although their origin and the mechanism of their release have not been completely elucidated.

The reasons for the high stability of circulating miRNAs remain largely unknown as well, although several hypotheses have been suggested [9]. First, circulating miRNAs might have unique modifications, such as methylation, adenylation, and uridylation that increase their stability and thus protect them against RNAses [10,11]. Second, circulating miRNAs might be protected by the encapsulation in the cell-derived microvesicles [12] or by specific RNA-binding proteins [13].

Despite the little information about the biogenesis and stability of circulating miRNAs, a little bit more is known about their functional role in determining a biological effect even in distant sites of the body. In fact, three different hypotheses explaining the origin and stability of circulating miRNAs in body fluids have been suggested [9]. The first suggests a passive release of miRNAs from apoptotic/necrotic cells and injured tissues, chronic inflammation, or from cells with a short half-life, such as platelets. The second suggests an active secretion via cell-derived microvesicles, including exosomes and shedding vesicles, which are membrane-enclosed cell fragments released by cells under both normal and pathological conditions [14]. Exosomes are formed via the inward budding of early endosomal membranes, giving rise to intracellular multivescicular bodies that later fuse with the plasma membrane and are released to the extracellular environment. Shedding vesicles are larger vesicles that are generated by the outward budding and fission of the plasma membrane. The loading of miRNAs into microvesicles seems to be controlled by specific proteins of the RNA-inducing silencing complex [15]. The last hypothesis involves the active secretion by cells of miRNAs and RNA-binding protein complexes. Some of these RNA-binding proteins are represented by nucleophosmin (i.e., the nucleolar phosphoprotein NMP1) [9], high/low density lipoproteins (LDL) [16] and Argonaute 2 (Ago2) [12]. In a recent study, the release of miRNAs in the bloodstream was shown to be controlled by neutral sphingomyelinase 2 and through ceramide-dependent secretory machinery [16]. These results suggest the existence of a specific miRNA export system by which certain miRNAs are exported by some cells, but are recognized, taken up and employed by others.

## 3. Circulating miRNAs and Toll-Like Receptors

Another open question is how circulating miRNAs are taken up by recipient cells, that is, whether this process occurs randomly (passive transport) or involves the targeting of specific cells (active transport) [9]. It has been postulated that microvesicle-enclosed miRNAs could be internalized by recipient cells through endocytosis, phagocytosis, or direct fusion with the plasma membranes, whereas RNA-binding, protein-associated miRNAs could be taken up via specific receptors on the recipient cell surface. Alternatively, specific antigen molecules on the microvesicle surface could be recognized by specific cell receptors. Recent studies highlighted the importance of extracellular miRNAs in cell-to-cell communication and suggested a possible mechanism of intercellular transfer of genetic information [12,17]. The identification of circulating miRNAs released by cells within exosomes and the demonstration of their ability to be transferred from one cell to another, provocatively suggested a functional comparison of circulating miRNAs to hormones. Following this hypothesis, “hormone-like” miRNAs should also have a proper receptor to bind with. Among the many candidate receptors we may think of, the toll-like receptor (TLR) family is particularly appealing for several reasons. First, TLRs are a family of receptors through which the mammalian innate immune system recognizes the presence of invading pathogens [18]. It has been recently demonstrated that members of the TLR family (i.e., murine TLR7 and human TLR8) on dendritic cells and B lymphocytes can recognize and bind viral, single-stranded RNA (ssRNA) sequences, leading to cell activation and the production of cytokines [19]. Both murine TLR7 and human TLR8 are activated by the binding to a 20-nt long ssRNA, a physiologic ligand for these two receptors. Therefore, as circulating miRNAs have a comparable length, they could represent endogenous agonists of these TLRs. Fabbri and colleagues observed that the pro-inflammatory and pro-metastatic responses in human cancers are triggered by two circulating miRNAs, miR-21 and miR-29a, and that this response is mediated only by TLR8. This is a finding that prompts further studies aimed at understanding the selective role of TLRs, a necessary background in order to properly develop selective and specific TLR7- (or TLR8-) binding anticancer drugs [20]. Interestingly, the implications of circulating miRNAs binding to TLRs are not disease specific and go beyond cancer. In fact, Lehmann and colleagues showed that let-7b, a key regulator of gene expression in the central nervous system, is expressed at higher concentration in the cerebrospinal fluid of patients with Alzheimer’s disease. The exogenous intrathecal injection of let-7b into the cerebrospinal fluid of wild-type mice resulted in neurodegeneration, whereas mice lacking TLR7 were resistant to this neurodegenerative effect. However, the susceptibility to let-7b was restored in neurons of TLR7-/- fetuses transfected with TLR7 through intrauterine electroporation [21]. These findings emphasize that in addition to miR-21 and miR-29a, let-7b can also bind to TLR7, and indicate that other circulating miRNAs may have a role in mediating the activation of different processes (i.e., pro-metastatic inflammatory response) leading to different diseases [22].

## 4. Quantification and Analysis of Circulating microRNAs

The expression of miRNAs can be easily determined by various methods (i.e., quantitative polymerase chain reaction (qPCR), microarrays and next generation sequencing) [23,24,25,26,27,28]. Maybe, one of the most powerful methods for the analysis of circulating miRNAs is qPCR which in some applications involves the use of a reverse transcription PCR (RT-PCR) stem-loop primer [29]. This method can also in principle be employed for the detection of poorly expressed circulating miRNAs although its practical use is debated [30]. Microarray profiling of circulating miRNAs is a technique largely employed in those studies that allow an amount of starting material greater than the conventional amount used for qRT-PCR. Moreover, the need to develop updated probes and dedicated hybridization conditions for the detection of different miRNAs concurrently, has somehow hindered the use of this technique [5]. Recently, next generation sequencing (deep sequencing) appeared to be a very promising technique for the identification of novel miRNA biomarkers [31,32,33]. This technology has been used to identify tissue and stage-specific mRNA and miRNA expression, and to compare miRNA profiles in different diseases [31]. This method seems also quite suited for the quantification of circulating miRNAs, provided that particular care is put on sample preparation. We will describe just a couple of significant examples published in the last few years, but we are aware that many other papers are appearing in the literature and the list of applications is continuously growing. In the first paper, Williams et al. profiled cell-free miRNAs in serum and plasma samples from human volunteers by introducing synthetic oligonucleotides as calibrators during library preparation, thus being able to calculate the total as well as the specific concentration of circulating miRNAs [34]. The authors studied trios of samples (i.e., newborn babies and their parents) and were able to detect placental-specific miRNAs in both maternal and newborn circulations and to quantify the relative contribution of placental miRNAs to the circulating pool of miRNAs. This work can be therefore considered a model to be employed not only in the field of prenatal diagnosis but also to detect other diseases. As illustrated in previous paragraphs, circulating exosomes contain miRNAs that can be sequenced. Bellingham and coworkers performed the first small RNA sequencing (RNA-Seq) experiment to investigate the profile of miRNAs contained in exosomes released from prion-infected neuronal cells [35]. They demonstrated that neuronal exosomes contained a wide range of RNA species: retroviral RNA repeat regions, mRNA fragments, transfer RNAs (tRNAs), non-coding RNAs, small nuclear RNAs, small nucleolar RNA, other RNAs, and also novel candidate miRNAs. The results illustrated in this work clearly demonstrated that it is possible to obtain a “miRNA signature” from exosomes and that this signature can be potentially exploited not only to better understand the pathogenesis of these diseases but also for their diagnosis. The sequencing of circulating miRNAs has also been exploited to identify novel markers predicting clinical outcome for locally advanced breast cancer [36]. In fact, the authors used a comprehensive de novo sequencing approach and identified specific sets of miRNAs that were associated with various histopathological parameters, and two sets (miR-375 and miR-122) associated with clinical outcomes (higher levels of these miRNAs are associated with metastatic recurrences) in breast cancer patients (stage II–III). These results are noteworthy and may allow optimized chemotherapy treatments and preventive anti-metastasis interventions in clinical applications.

However, one of the main issues when dealing with the quantification of circulating miRNAs is the reproducibility among different platforms [37] or different methods (i.e., microarrays or RT-PCR arrays) [7] and different protocols for sample preparation [38] and miRNAs isolation [39]. Moreover, the statistical data analysis is also inevitably different from the classic approach. In fact, one of the most common problems is which normalization strategy to adopt after performing qPCR profiling [40]. So far, several normalization strategies have been used for the analysis of circulating miRNAs. Initially, some authors employed calibrators classically used for mRNA normalization of qPCR data (i.e., GAPDH, RNU6B, 18S, 5S) [41,42,43], but their variable or absent expression in circulation and their possible degradation prevented their further use [44]. Other strategies involved the use of synthetic *C. elegans* miRNAs, such as cel-miR-39 and cel-miR-54, two species without homology with human miRNAs, as spike-in and normalization controls [45,46]. However, since they are not endogenous miRNAs, their use is not methodologically recommended. To prevent this limitation, constitutively expressed miRNAs (i.e., miR-16) have been used as an internal normalization control [47,48]. Although quite stable and highly expressed in many conditions, this miRNA is not suitable for this purpose since it has been found modulated in many diseases and in tumors [49] and its expression level is hemolysis-dependent [50]. To circumvent all these problems, Mestdagh et al. introduced the global mean normalization method to normalize qPCR miRNA profiling data [51]. In fact, in these studies a large number of miRNAs are evaluated at the same time and the use of this normalization strategy has been demonstrated to outperform other normalization strategies [51]. Other normalization strategies can be adopted (i.e., the use of other internal normalizers such as miR-103a-3p or miR-191-5p) or even other techniques [52], although there is not a consensus as to the optimal normalization strategy, especially in the pediatric setting.

## 5. Circulating microRNAs in Biological Fluids

**Figure 2:**
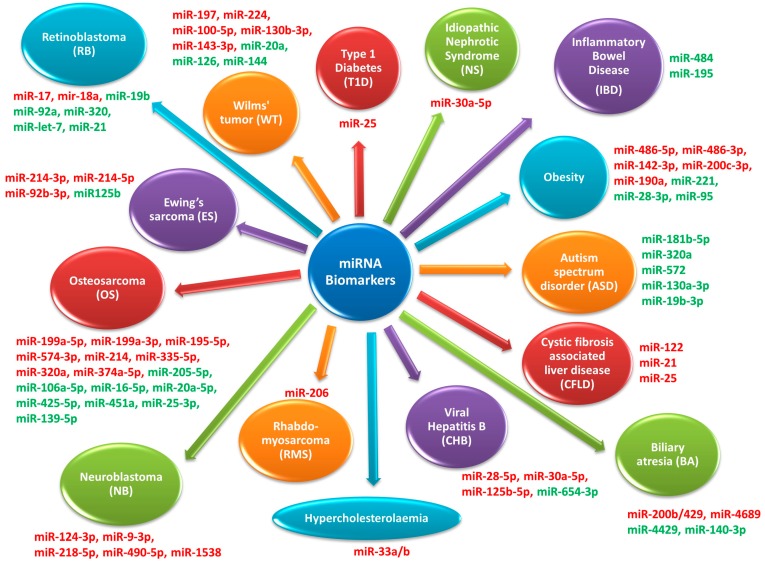
Circulating miRNAs detected in different pediatric diseases. Up- and down-regulated miRNAs are represented in red and green, respectively.

In the last few years, much evidence emphasized that some of the miRNAs previously identified in cells and tissues were also present in extracellular biological fluids such as serum, plasma, saliva and urine [8,9,53]. It has been observed that the expression level and the profile of these extracellular miRNAs are closely correlated with the disease or its progression [54,55]. These findings suggested that extracellular miRNAs could be employed as informative biomarkers of different diseases. With the aim of collecting and discussing the recent papers describing the use of circulating miRNAs as biomarkers in pediatric diseases, we considered many common diseases such as type 1 diabetes, idiophatic nephrotic syndrome, Crohn’s disease, ulcerative colitis, obesity, autism spectrum disorder (ASD), cystic fibrosis-associated liver disease (CFLD), biliary atresia, viral hepatitis B, hypercholesterolemia and pediatric solid tumors. In particular, we specifically discuss those studies where circulating miRNAs have been found not only in biological fluids such as serum and plasma, but also in urine, in order to explore the feasibility of alternative sampling sources (different from serum/plasma) for non-invasive pediatric diagnostics (Figure 2). Moreover, we briefly discussed miRandola, a novel database integrating some bioinformatics tools (i.e., miRò, miRiam and miRScape) and links to other useful tools/databases for the annotation and functional analysis of circulating miRNAs [56].

## 6. Circulating MicroRNAs in Plasma/Serum

### 6.1. Type 1 Diabetes 

Type 1 diabetes (T1D) is an autoimmune disease which originates from an immune-mediated damage of the insulin-producing beta cells and the appearance, in circulation, of pancreatic autoantibodies [57]. This cell death implies a progressive, irreversible loss of the endogenous insulin production, leading patients to daily treatment with exogenous insulin. At the time of diagnosis, a child with T1D is estimated to have lost approx. 80–90% of the insulin-producing beta cell function/mass [57]. Soon after the initial insulin treatment, several children experience a period of increased endogenous insulin production followed by a reduced need of exogenous insulin, referred to as the remission phase [57]. The regenerative potential that exists within this time interval, particularly in children, gives a unique possibility of intervention treatment. From a diagnostic point of view, Nielsen et al. compared the circulating miRNA levels in children affected by T1D (approx. 400 individuals) compared to controls (150 healthy age-matched children). Twelve differentially expressed miRNAs between cases and controls were identified. Several of these miRNAs are involved in the regulation of apoptosis (miR-181a, miR-24, miR-25, miR-210, and miR-26a) and beta-cell regulatory networks (miR-24, miR-148a, miR-200a, and miR-29a) [58]. Two other miRNAs (miR-152 and miR-30a-5p) have been found deregulated in T1D, but their function is still a debated issue. Interestingly, an association between miR-25 and better glycemic control and residual beta-cell function has been found. This association suggests that miR-25 could be an important biomarker for the early diagnosis of diabetes, to improve blood glucose control and reduce microvascular complications. This study can serve as a proof-of-concept study where the use of circulating miRNAs has been demonstrated as a clinically relevant diagnostic tool, and also useful to evaluate the proper therapeutic interventions focused on preserving/regenerating beta-cell function in T1D patients.

Another study investigated the circulating miRNA profiles of sera from children with recent-onset T1D [59]. Interestingly, the authors found 35 significantly modulated miRNAs compared to the controls, 27 of which were upregulated in T1D. Six miRNAs (miR-24-3p, miR-140-5p, miR-144-5p, miR-222-3p, miR-345-5p and miR-454-3p) were elevated specifically at early and not at later stages of diabetes. Therefore, this study allowed the identification of a miRNA pattern that may be also involved in T1D pathogenesis.

### 6.2. Idiopathic Nephrotic Syndrome

Childhood nephrotic syndrome (NS) is the most frequent glomerular disease in childhood, generally caused by a functional impairment of the immune system. This disease is characterized by alterations in permselectivity at the glomerular capillary wall, which leads to an increase of protein secretion in urines [60]. NS is estimated to affect 2–7 per 100,000 children, with a prevalence from 12 to 16 per 100,000 individuals. The clinical hallmarks of NS are heavy proteinuria, edema, hypoalbuminemia, and hyperlipidemia [61,62]. If untreated, NS may lead to an increased risk of life-threatening infections, thromboembolism, lipid abnormalities, and malnutrition [63]. However, more than 80% of children with idiopathic NS are well responsive to the treatment with prednisone [64].

In a small number of patients with lupus erythematosus [65], Immunoglobulin A nephropathy (IgAN) [66,67] and acute or chronic renal injury [68,69] the authors reported a dysregulation of circulating or urinary miRNAs and suggested that miRNAs in body fluids may help to diagnose human diseases and may be employed as a novel class of non-invasive disease biomarkers.

In a recent paper, Luo and coworkers reported for the first time the serum and urinary miRNA profile in patients affected by NS [70]. Sera and urine from NS patients (*n =* 33) and controls (*n =* 30) were separately pooled and analyzed by qPCR (TaqMan low density arrays). Statistical analysis identified five miRNAs (miR-30a-5p, miR-151-3p, miR-150, miR-191, and miR-19b) in the serum of NS patients compared to controls. Logistic regression analyses and Receiver Operating Characteristic (ROC) curves revealed a strong correlation between the dysregulated miRNAs and NS. Of note, only miR-30a-5p has been found also to be statistically upregulated in the urine of NS patients compared to controls, and to be positively correlated to the serum level. These data will afford not only the obtaining of novel biomarkers for diagnosis and the assessment of pediatric NS, but could also provide novel functional insight into the pathogenesis/progression of the disease. In fact, the pathogenesis of idiopathic childhood NS is still unknown. Recent evidence suggests that the primary defect in NS is at the level of glomerular visceral epithelial cells (podocytes), which are key cells in the selective filtering action of the glomerular capillary wall [71]. Recently, several studies performed on mice models have demonstrated the important role of miRNAs in the development and function of podocytes and glomeruli [72,73]. The authors demonstrated that during development, the podocyte-specific deletion of Drosha or Dicer, two key enzymes that function in a stepwise manner to generate mature miRNAs, led to proteinuric renal disease or multiple abnormalities such as podocyte apoptosis and depletion, and glomerulopathy. The authors found that the mutant phenotype is due to the loss of miRNAs and that miRNAs are required for the normal function of mature podocytes. Recent findings have suggested that miR-30a may have a role in the homeostasis and podocytopathies of podocytes [74]. Dicer-knockout mouse podocytes cannot synthesize miR-30a and mutated mice developed proteinuria by three weeks after birth. Moreover, they progressed rapidly to end-stage kidney disease. Therefore, miR-30a seems to be an important genomic regulator of molecular podocyte homeostasis and could be involved in the pathogenesis of childhood NS. These results suggest once more that circulating miRNAs not only play an important role in the pathogenesis and progression of pediatric NS, but also hold promises as novel diagnostic and prognostic indicators of childhood NS.

### 6.3. Inflammatory Bowel Disease

Inflammatory bowel disease (IBD) is a multi-factorial inflammatory disorder of the gastrointestinal tract and includes other diseases such as Crohn’s disease (CD) and ulcerative colitis (UC). Both CD and UC are characterized by chronic inflammation of the gastrointestinal tract that may arise as a consequence of an aberrant immune response to gut microbiota in genetically predisposed individuals. The specific role of miRNAs in intestinal diseases are not well understood. In mouse models, the loss of intestinal miRNAs has been demonstrated to impair epithelial barrier function, resulting in acute inflammation [75]. Moreover, little is known about the interplays between gut microbiota and gene expression regulation by miRNAs [76] although a recent paper has suggested a potential mechanism of host–guest inter-kingdom communication [77]. In CD, the alteration of disease-specific circulating miRNA levels has been demonstrated to correlate with epithelia damage and inflammation [78]. In particular, authors found that circulating miR-192 is elevated in CD and that this microRNA is greatly expressed in intestinal epithelia [78]. Moreover, the authors examined the diagnostic properties of circulating miRNAs compared to some standard serological markers (i.e., C-reactive protein (CRP), anti-*Saccharomyces cerevisiae* antibodies (ASCAs), Immunoglobulin G (IgG), and albumin) and they found that miR-484 and let-7b displayed a sensitivity of 80%, whereas the use of three microRNAs (miR-30e, miR-160a and miR-195) showed a sensitivity greater than 90%. To exclude that these miRNAs could represent unselective indicators of intestinal inflammation conditions, the authors compared the expression of circulating miRNAs in patients with celiac disease. In this latter case, the expression level of these miRNAs remained unaltered, thus confirming their specificity for IBD or CD. Moreover, the expression level of circulating miR-484 and miR-195 reduced after six months of treatment (systemic steroids 16 (67%), methotrexate 1 (4%), 6-mercaptopurine or azathioprine 10 (42%), and infliximab 2 (8%), suggesting that these miRNAs could be employed not only as diagnostic biomarkers but also as indicators of the outcome of therapeutic interventions. For the above-mentioned diseases, circulating miRNAs have been considered more versatile biomarkers compared to serological ones.

Duttagupta has investigated the expression of circulating miRNAs within microvesicles, peripheral blood mononuclear cells and platelets, in a cohort of 20 UC patients and 20 normal individuals [79]. A distinct diagnostic signature of 31 miRNAs was isolated, with increased expression from microvesicles to platelets. The platelet miRNA profile can stratify UC patients from normal individuals with 92.8% accuracy, 96.2% specificity and 89.5% sensitivity. The authors observed a significant overlap between the miRNA profiles from the platelet-only fraction and the platelets and microvesicles fraction [79]. However, there were poor performance metrics for the non-platelet miRNAs. Therefore, Duttagupta and collaborators concluded that the origin of the majority of circulating miRNAs in UC derive from the anucleate platelet fraction.

A few years ago, Zahm suggested that circulating miRNAs could be used as potential biomarkers of colitis in adolescents with IBD [80]. The authors compared the expression of miRNAs in 50 mucosal biopsies with the correspondent serum miRNA profiles. Several miRNAs have altered expression in the biopsies of IBD patients and controls, but only miR-142-3p and miR-21 were among the most abundant together with miR-192 and miR-194. MiR-192 and miR-21, two circulating miRNAs previously associated with pediatric CD, displayed high expression level in IBD samples compared to controls, but were not significantly different between the two disease subgroups (CD and UC). MiR-192, miR-142-3p, and miR-21 correctly classified 78.72%, 72.34%, and 72.34% of patients, respectively, but serum miRNA levels did not significantly correlate with tissue miRNA. However, the study revealed that no miRNAs were significantly altered between groups and the authors concluded that serum miRNAs are not useful biomarkers to distinguish UC from CD in adolescents [80].

Celiac disease is a severe intestinal autoimmune disease that has been widely investigated for its increasing prevalence and important social and economic implications. So far, little is known about the presence of reliable circulating microRNA biomarkers to diagnose the disease or to monitor the progression or remission after a gluten-free diet. In order not to lengthen the present review with a detailed description of the studies dealing with this important pediatric disease, we collected and discussed the papers about circulating microRNAs and celiac disease in a recent paper [81].

### 6.4. Obesity

Obesity is a well-known epidemic health problem worldwide. Obese patients suffer from decreased life quality and expectancy, as well as increased risk of type 2 diabetes, cardiovascular diseases, hepatic steatosis, and cancer [82]. Body composition is likely determined by genetic makeup in close relationship with behavioral and environmental factors. The intake of energetic food combined with a reduced physical activity contributes to the high prevalence of obesity. Many efforts have been made to identify obesity-related genes that should allow us to better understand its pathogenesis and find novel targets for clinical therapy. Moreover, they should also allow early prediction of metabolic complications. Actually, new tools such as high-throughput sequencing may contribute to solving several problems in clinical practice, and allow an earlier and more accurate diagnosis of comorbidities and improve prediction and response to therapy [83]. Can et al. have evaluated the association between circulating miRNA levels and lipid metabolism in obese and non-obese children and adolescents [84]. They found that circulating miR-370, miR-33, miR-378 and miR-27 were increased, and circulating miR-335, miR-143 and miR-758 were decreased in obese children. They asserted that low levels of miR-335, miR-143 and miR-758, and high levels of miR-27, miR-378, miR-33 and miR-370 might be responsible for the elevated levels of cholesterol, triglycerides (Tg) and LDL and low levels of high density lipoprotein-cholesterol (HDL-C) in obese subjects [84]. Prats-Puig provided the first evidence that circulating miRNAs are deregulated in childhood obesity and that concomitant variations during growth are different in children who tend to gain or lose weight [85]. The cross-sectional validation study revealed that fifteen specific circulating miRNAs were significantly deregulated in pre-pubertal obesity. These miRNAs also include the downregulated miR-28-3p and miR-221 and the upregulated miR-130b, miR-142-3p, miR-423-5p, miR-486-5p and miR-486-3p (*p*-value < 0.0001). The amount of these circulating miRNAs was significantly correlated with the body mass index (BMI) and other parameters (i.e., waist, percent fat mass and regional fat distribution) or with other laboratory parameters such as homeostasis model assessment of insulin resistance, high-molecular-weight adiponectin, C-reactive protein, and circulating lipids. Ten circulating miRNAs changed their expression significantly during the follow-up period (three years) in children who increased or decreased their weight. Thus, miR-221, miR-28-3p, miR-142-3p, miR-486-5p, and miR-486-3p were validated as novel biomarkers for risk estimation and classification of obesity in children [85]. In a recent study, we revealed for the first time the significant associations between selected circulating miRNAs and insulin resistance (IR) in obese preschoolers [86]. Associations were investigated at fasting and after glucose load (oral glucose tolerance test, (OGTT). Our study identified circulating miR-200c-3p, miR-190a and miR-95 as biomarkers of insulin resistance in obese preschoolers, as they were differentially regulated in IR patients both in fasting condition and after the OGTT. The expression behavior of some circulating miRNAs seems to reflect the glucose and insulin excursion following the OGTT in a quite different way between the controls and IR obese preschoolers [86].

Circulating miRNAs related to endothelial dysfunction, which is another obesity-related disease, have been recently investigated [87]. The authors concluded that circulating miRNAs may be used as powerful screening tools for assessing endothelial dysfunction in children, or to identify endothelial-dysfunction-relevant target genes. Similarly, in pediatric obstructive sleep apnea (OSA), a unique subset of circulating miRNAs has been discovered to be involved in OSA and endothelial dysfunction [88].

### 6.5. Autism Spectrum Disorder

Autism spectrum disorder (ASD) refers to a group of heterogeneous neurodevelopmental disorders characterized by an impairment of communication and social interactions, and restricted, repetitive and stereotypical patterns of behavior [89]. ASD has mainly a genetic origin, with most data supporting a polygenic model [89,90]. However, because of the heterogeneity of this disorder, classical genetics has not been able to identify suitable candidate genes for ASD. Moreover, environmental factors may also play a vital role in predisposing individuals to ASD [91]. In the past few years, some epigenetic mechanisms have been identified as potential contributors to the pathogenesis of ASD [92]. In fact, epigenetic factors may change gene expression without changing the DNA sequence [93]. Mundalil et collegues observed that ASD subjects have an altered expression of 13 serum miRNAs (eight miRNAs were downregulated and five upregulated). High sensitivity, specificity and area under the curve (AUC) values were observed for miR-181b-5p, miR-320a, miR-572, miR-130a-3p and miR-19b-3p. Therefore, these five miRNAs may be potential candidates for circulating miRNA-based prediction of ASD [94].

### 6.6. Cystic Fibrosis-Associated Liver Disease

CFLD is a well-known complication of CF that has become increasingly important and consists of a progressive hepatobiliary fibrosis that has a significant impact on morbidity and mortality [95]. The onset and progression of fibrosis in CFLD are difficult to predict and monitor, and factors related to the pathogenesis of CFLD have not been fully elucidated. The current diagnostic procedures for an early detection of CFLD and the stage of fibrosis severity are still inadequate. Liver biopsy remains the gold standard criterion for the diagnosis of hepatic fibrosis in CFLD, but this procedure is invasive and may be not conclusive [96]. Cook and colleagues demonstrated that the combination of three circulating microRNAs (miR-122, miR-21, and miR-25) can be used to classify CF patients with liver diseases. Furthermore, by using different miRNAs they were able to discriminate CFLD patients in different groups according to no fibrosis (F0), severe fibrosis (F3–F4) and those with any histological evidence of fibrosis (F1–F4). In particular, the combined use of serum miR-210, miR-148a, and miR-19a allowed the detection of early liver fibrogenesis (F0–F1) and noninvasively discriminate CFLD children in two groups, with (F1–F4) or without (F0) fibrosis [97].

### 6.7. Biliary Atresia 

Biliary atresia (BA) is an idiopathic neonatal liver disease characterized by inflammatory and fibrotic obliteration of the extrahepatic bile ducts, leading to severe cholestasis and biliary cirrhosis [98]. The etiology of BA is still an unresolved issue. BA is thought to be caused by viral infections, exposure to toxins, defects in morphogenesis, genetic predisposition, and immune dysregulation. Patients with BA suffer from progressive liver fibrosis and cirrhosis, and rarely survive longer than two years without treatment [98]. Zahm and colleagues have identified a BA-specific circulating miRNA signature that may serve as a novel diagnostic tool. The screening of serum miRNAs identified nine miRNAs that are significantly altered in BA compared to cholestatic controls. Subsequent validation in a larger, independent cohort confirmed that a single miRNA cluster (i.e., miR-200b/429) was significantly increased in BA patients. ROC analysis emphasized the diagnostic utility of miR-200a, miR-200b and miR-429 as they correctly diagnosed up to 85% of patients. However, they did not demonstrate whether a linear combinatory model of these microRNAs could have improved diagnostic accuracy [99]. Dong et al. studied 45 BA children and 20 non-BA cholestatic children as controls [100]. A number of 13 differentially expressed miRNAs were identified (two up-regulated and 11 down-regulated) and eight miRNAs were further selected for qRT-PCR validation on an independent set of children with BA (*n =* 35) and controls (*n =* 20). Four miRNAs including hsa-miR-150-3p, hsa-miR-4429, hsa-miR-4689 and hsa-miR-92a-3p were differentially expressed. The validation of these miRNAs showed a significant down-modulation of miR-4429 and an upregulation of miR-4689 in the BA group. The ROC curves afforded an area under the curve (AUC) of 0.789 and 0.722 for miR-4429 and miR-4689, respectively. The authors suggested that these values may show promising diagnostic performance [100]. Interestingly, the miRNAs reported by Zahm et al. [99] were not found dysregulated in the Dong et al. study, probably due to the small sample size in the analysis (*n =* 4 per group) or to differences in the selected cohorts. However, these recurrent differences between reported miRNA expression profiles emphasize the need for a standardized methodological approach and study design that include prospective validation in large-scale studies. Another study by Peng and coworkers was the first to characterize circulating miRNAs in patients with BA using next-generation sequencing [101]. The authors identified a distinct plasma profile of 15 miRNAs that may be involved in the onset and progression of BA. Of these, seven were upregulated (let-7c-5p, miR-100-5p, miR-122-5p, miR-194-5p, miR-200a-3p, miR-432-5p and miR-574-5p) and eight downregulated (miR-10b-5p, miR-23a-3p, miR-26a-5p, miR-126-3p, miR-140-3p, miR-142-3p, miR-370-3p and miR-744-5p). A second set of 44 BA patients, 20 non-BA cholestatic disease patients and 20 healthy controls was used to validate the Next Generation Sequencing (NGS) findings. The validation phase showed an increase in the expression level of miR-100-5p and miR-122-5p and a decrease of miR-126-3p and miR-140-3p in BA patients. Moreover, BA patients showed a significant decrease of miR-140-3p when compared to cholestatic patients. Strikingly, decreased levels of plasma miR-140-3p distinguishes BA from other neonatal cholestatic disorders (AUC = 0.75). Moreover, the expression level of miR-200a found in this work confirmed the findings of the previous work by Zahm [99].

### 6.8. Viral Hepatitis B

Chronic hepatitis B (CHB) is a global health problem, with more than 350 million people chronically infected worldwide [102]. Children are generally infected during birth or infancy, with infections persisting in 90% of cases. Approximately 5% of these develop chronic diseases. CHB in children is reported to be associated with a 25% risk of severe adverse outcomes such as cirrhosis and hepatocellular carcinoma [102]. Hepatitis B virus (HBV) is not cytopathogenic and liver damages are generally due to the host immune system. The natural course of CHB is characterized by three stages: immune tolerance, active, or inactive stages [103]. Most of the children have an immune-tolerant stage, a high viral load, a measurable hepatitis B e-antigen (HBeAg) and slightly elevated alanine aminotransferase (ALT). Children in the immune inactive stage have a lower risk of liver disease progression but a reactivation of hepatitis B virus may occur [103]. Winther et al. were the first to investigate the plasma miRNA profile of children with CHB. A group of 16 miRNAs (miR-99a, miR-100, miR-122, miR-122*, miR-125b, miR-192, miR-192*, miR-193b, miR-194, miR-215, miR-365, miR-455-5p, miR-455-3p, miR-483-3p, miR-885-5p, and miR-1247) was identified as significantly and differentially expressed in HBeAg positive, HBeAg negative and healthy children groups. Of note, all of the miRNAs, with the exception of miR-1247, are associated with CHB in adults [104,105,106], substantiating the importance of these miRNAs in the pathogenesis of pediatric CHB. The differentiation between the presence and absence of HBeAg is of great importance for children with CHB. The authors demonstrated that all of the 16 identified miRNAs were highly upregulated in HBeAg-positive children compared with HBeAg-negative ones. A strong positive correlation was found between the plasma levels of the identified miRNAs and the amount of HBV DNA. This data indicates the existence of a relationship between the abundance of circulating miRNAs and the immunological stages in the natural course of the disease. Some of these miRNAs may contribute to the establishment and maintenance of CHB in children [107]. In another study, Winther et al. revisited their previous screen of plasma microRNA levels in HBeAg-positive and HBeAg-negative children with chronic hepatitis B (CHB) and in healthy controls. Several candidate microRNAs with aberrant plasma expressions in HBeAg-positive children were identified. Thirteen microRNAs (miR-28-5p, miR-30a-5p, miR-30e-3p, miR-125b-5p, miR-193b-3p, miR-215, miR-365a-3p, miR-378a-3p, miR-455-5p, miR-455-3p, miR-574-3p, miR-654-3p, and miR-let-7c) showed aberrant plasma expressions in HBeAg-positive children and targeted liver-specific genes. In particular, three microRNAs were upregulated (miR-28-5p, miR-30a-5p, and miR-125b-5p) and one was downregulated (miR-654-3p) in HBeAg-positive children compared to HBeAg-negative and healthy control children, who showed equal levels. They proposed that these microRNAs identified with aberrant plasma expressions specifically in HBeAg-positive children and with liver-specific target genes that are biomarkers for disease progression and might impact the development of hepatocellular carcinoma (HCC), and perhaps also cirrhosis, in children with CHB [108]. In a third study, Winther et al. characterized microRNA plasma levels in the natural history of CHB in children. They demonstrated that the circulating levels of four miRNAs (miR-99a-5p, miR-122-5p, miR-122-3p, and miR-125b-5p) decrease significantly over time in immune-tolerant and immune-active children, whereas the miRNA plasma levels are stable in immune-inactive children. This is the first study aimed at characterizing the plasma microRNAs and HBsAg over time in children with CHB. These data suggest that the plasma levels of selected microRNAs and HBsAg are inversely correlated with immunological control of CHB in children. Further studies are, however, needed to advance the understanding of microRNAs and HBsAg in the pathogenesis of CHB in children [109].

### 6.9. Hypercholesterolemia 

Hypercholesterolemia is one of the major causes of cardiovascular diseases (CVD). Hypercholesterolemia is associated with enhanced oxidative stress, which leads to increased lipid peroxidation, which in turn determines endothelial dysfunction, susceptibility to coronary vasoconstriction and ultimately atherosclerosis. In children with hypercholesterolemia (HC), early functional and anatomic changes of the arterial wall have been detected [110]. Moreover, it has been observed that the flow-mediated dilatation (FMD) was decreased, suggesting a role for cholesterol in impairing nitric oxide (NO) release or inhibiting endothelium NO synthase [111]. Different miRNAs are involved in the pathogenesis of CVD. Together with atherogenic factors, they play a crucial role in the control of the inflammatory process and they can stimulate the atherosclerotic degeneration of the vessel walls of arteries. Martino, in his study, found that miR-33a/b was significantly up-regulated in the plasma of children with hypercholesterolemia and positively correlated with the levels of TC, LDL-cholesterol, ApoB, CRP and glycaemia [112]. Thus miR-33a and miR-33b up-regulation could play an active role in the pathogenesis of CVD that has been already described in these children, opening a new window for a therapeutic intervention for this disease. miR-33a and miR- 33b could also be employed as new prognostic markers and/or as effective therapeutic targets for CVD associated with pediatric hypercholesterolemia.

### 6.10. Solid Tumors

Pediatric cancers represent 2–3% of all malignancies and are a major cause of death for affected children and adolescents [113]. Pediatric cancers can be classified into two groups: solid tumors and blood cancers. Pediatric solid tumors are different from adult solid tumors, as there are some solid tumors that occur in children but never develop in adults. The most common types of solid tumors include neuroblastoma, rhabdomyosarcoma (RMS), Ewing’s sarcoma (ES), osteosarcoma (OS), retinoblastoma (RB) and Wilms’ tumor (WT) [114].

Many studies have investigated circulating miRNAs to identify novel biomarkers for non-invasive diagnosis and follow-up. The profile of miRNA in biofluids may differentiate different types of tumors [115]. In a recent study, Murray and coworkers recruited 20 healthy donors and 34 patients (aged 0–16) at the time of malignant tumor diagnosis. Patients included (i) four neuroblastoma (NB) comprising two N-myc proto-oncogene protein (MYCN)-amplified INRG (International Neuroblastoma Risk Group) high-risk tumors and two non-MYCN-amplified INRG low-risk tumors; (ii) four hepatoblastomas; (iii) seven WT; (iv) seven lymphomas comprising five cases of classical nodular-sclerosing Hodgkin’s disease and two cases of B-cell non-Hodgkin’s lymphoma; (v) six sarcomas comprising three RMS, two ES, one OS; (vi) one *DICER1*-mutated pleuropulmonary blastoma and (vii) five central nervous system tumors comprising; three WHO (World Health Organization) 2007 grade I, one grade II and one grade III gliomas. Through qPCR, five overexpressed miRNAs (miR-124-3p, miR-9-3p, miR-218-5p, miR-490-5p and miR-1538) have been validated in high risk NB compared to other tumors and controls; three overexpressed miRNAs (miR-214-3p, miR-214-5p and miR-92b-3p) have been validated in ES, compared to RMS and OS; and three overexpressed miRNAs (miR-500a-5p, miR-512-5p and miR-519a-3p) have been validated in OS compared to RMS and ES [115].

Myiachi investigated circulating biomarkers in RMS patients (*n =* 10), in other pediatric tumors (*n =* 28) and healthy children (*n =* 17) [116]. The expression of muscle-specific miRNAs (i.e., miR-1, miR-133a/b and miR-206) were significantly higher, especially miR-206, in patients with RMS than in other tumors or in the control group, suggesting the possible use of circulating miRNAs as potential biomarkers of RMS.

Osteosarcoma is the most common human primary malignant bone tumor in children and young adults and Liu Ouyang and colleagues investigated the expression profiles of a panel of miRNAs for diagnostic purposes [117]. Six miRNAs (i.e., miR-21, miR-199a-3p, miR-143, miR-34, miR-140, and miR-132) were assessed by qPCR in the plasma of 40 OS patients and 40 healthy controls. The circulating levels of miR-21 significantly increased in patients with OS compared to controls, whereas miR-199a-3p and miR-143 decreased. ROC curve analysis demonstrated that these three-miRNA signature could efficiently discriminate cases from controls. In another study, the levels of miR-34a, miR-34b, and miR34c, a tumor suppressor miRNA family that is transcriptionally activated by p53, were detected both in the plasma and tissues samples of 133 OS patients and 133 healthy controls [118]. Quantitative polymerase chain reaction analyses showed that the plasma and tissue miR-34b levels were significantly lower in the OS patients compared to controls. Yuan and colleagues, given the relationship of miR-21 with malignancies, measured its levels in the serum of 65 patients with OS and 30 healthy controls by qPCR [119]. They found that the levels of miR-21 were significantly higher in patients with OS than in control subjects and correlated its expression with an advanced Enneking stage and to chemotherapy resistance. The expression of another miRNA implicated in several human solid malignancies was investigated by Wanli Ma and colleagues [120]. They detected the expression levels of circulating miR-148a in 19 OS patients who successfully underwent surgical resection, and of 89 healthy individuals. They found that miR-148a was significantly upregulated in OS patients compared to healthy controls. In another paper, Zhang and colleagues investigated by qPCR the expression of muscle-specific miRNAs (miR-133b and miR-206) in the sera and bone tissue of 100 patients with OS and 100 healthy controls [121]. The expression levels of miR-133b and miR-206 were significantly downregulated in osteosarcoma tissues and sera, whereas miR-196a and miR-196b were upregulated in OS patients.

The first miRNA profiling of 168 unique human miRNAs was performed on 20 pre-therapy OS patients and 20 healthy individuals, and the differentially expressed miRNAs were further validated by RT-qPCR [122]. Only miR-106a-5p, miR-16-5p, miR-20a-5p, miR-425-5p, miR-451a, miR-25-3p and miR-139-5p were downregulated in the serum of OS patients when compared with healthy controls, and ROC curve analyses indicated that these seven miRNAs were able to efficiently discriminate between OS patients and the healthy cohort. By using a different technique (i.e., TaqMan low-density qPCR array), Zhou and collaborators assessed the expression of circulating miRNAs in 60 OS patients before surgery, 28 patients after one month of surgery, and 60 healthy individuals, and they found that miR-199a-5p was significantly higher in OS patients than in controls [123]. In a similar study, Lian et al. examined 90 OS patients before surgery, 50 patients after one month of surgery, and 90 healthy individuals. They found four miRNAs (i.e., miR-195-5p, miR-199a-3p, miR-320a and miR-374a-5p) significantly increased in OS plasma before surgery that reversed their expression after the surgical intervention [124]. Another study of circulating miRNAs in 67 OS children resulted in the identification of miR-205-5p with decreased expression in patients with OS compared to healthy controls, whereas miR-574-3p, miR-214, and miR-335-5p increased [125]. The heterogeneity of the results of all of these studies is again one of the major drawbacks of this approach that should prompt researchers to find standardized experimental and bioinformatics techniques to analyze and interpret data.

The involvement of miRNAs in the progression of ES was suggested by the observation that miR-125b decreased in ES tissue [126]. Following this preliminary data, Nie and coworkers investigated the expression of this miRNA in the serum of ES patients [127]. The authors isolated total RNA from the sera of 63 children with ES and 126 healthy controls and they found significant downregulation of miR-125b in ES patients when compared with healthy controls. ROC curves also suggested that miR-125b can be a valuable biomarker for ES.

Many studies indicated that both tissue and circulating miRNAs are involved in RB pathogenesis and could be used as diagnostic, prognostic and therapeutic biomarkers [128], but only two studies have assessed the expression of circulating miRNAs. The first is by Beta and colleagues [129], who performed miRNA profiling on two serum samples: a pool of 14 children with RB and another pool of 14 controls. In an independent set of 20 children with RB and 20 normal individuals, they used qPCR to validate the upregulation of miR-17 and miR-18a and the downregulation of miR-19b and miR-92a. The second paper from Liu and colleagues assessed the expression of circulating miRNAs in 65 plasma samples from patients with RB and 65 healthy subjects [130]. The results indicated that the circulating levels of miR-320, miR-let-7e and miR-21 were downregulated in RB patients. Again, the heterogeneity of results points to a standardization of techniques, as already mentioned in regard to other works.

Nephroblastoma, also known as WT, is the sixth most common human childhood malignancy and the most common renal tumor in children, with the majority of cases generally occurring at ≈36 months. In a recent work, Schmitt investigated the circulating miRNA profiles of 43 WT patients [131]. Microarray results, validated by qPCR, showed the upregulation of miR-197 and miR-224 and the downregulation of miR-20a, miR-126 and miR-144*. Only miR-520d-3p were upregulated in the microarray experiments, but this was not validated with qPCR. In another study, Ludwig and colleagues identified circulating miRNAs in the sera of 32 WT patients before preoperative chemotherapy and 12 control subjects without malignant diseases [132]. The results obtained by qPCR showed that miR-100-5p, miR-130b-3p, and miR-143-3p have higher expression in WT patients compared to healthy individuals.

In conclusion, many studies dealing with the profile of circulating miRNAs have revealed their dysregulation and their potential impact in modulating important biological processes in pediatric diseases. However, many experimental challenges still remain to be solved, such as standardized methods and normalization strategies to improve miRNA signature accuracy, especially in biomarker discovery in oncology [52].

## 7. Bioinformatics Tools and Databases for Circulating Extracellular microRNAs

The growing evidence about the presence of circulating miRNAs and their role in cell–cell communication has outlined the need for suitable tools for collecting and rationalizing these data. To this aim, the first database named miRandola, for the classification of different extracellular/circulating miRNAs, has been developed [56]. MiRNAs are classified into four categories, based on their extracellular forms: miRNA-Ago2, miRNA-exosome, miRNA-HDL and miRNA-circulating. The database offers numerous pieces of information, including isolation and experimental methods, type of source samples, and associated diseases. Moreover, a direct link to the knowledge base “miRò” is provided to explore the biological functions of circulating miRNAs and their relationships with the phenotype [133]. Different tools embedded in miRandola allow users to search by mature miRNAs, miRNA family, sample, diseases and malignant cell lines, and potential biomarker role (Table 1). The results are directly linked to miRò for further functional annotations. It is noteworthy that users can also download the comprehensive database miRandola, which contains all the information about extracellular/circulating miRNAs or contribute to updating the database through an online data submission form. The current version of miRandola (last update February 2017) is focused on human circulating miRNAs, long non-coding and circular RNAs, integrates Vesiclepedia miRNA data, and is linked to the NIH exrna.org research portal. Moreover, miRandola can help to retrieve specific information about miRNA complexed with exosomes taken from the ExoCarta database, an exosome database that provides links to proteins, RNAs and lipids identified in the exosomes isolated from multiple organisms [134]. The new version of the ExoCarta database (Ver. 5; 29 July 2015) has more than two times the data compared to the last release in 2012 and has new additional features including the annotation of International Society for Extracellular Vesicles (ISEV) standards and dynamic protein–protein interaction networks. An integrated and comprehensive proteome, transcriptome, and lipidome database of EVs (extracellular vesicles) derived from archaea, bacteria, and eukarya, including humans is collected in EVpedia (Ver. 6 February 2015) that provides a search and browse tool for vesicular proteins, comparison of vesicular datasets by orthologue identification, gene ontology enrichment analyses and network analyses of vesicular proteins. Furthermore, EVpedia provides databases of vesicular mRNAs, miRNAs, and lipids [135]. FunRich—which provides functional enrichment and network analysis integrated with gene ontology, biological pathways, protein interactions, and domains or associated diseases—offers the opportunity to compare your proteomic data with ExoCarta [136]. FunRich also has an integrated plugin pertaining to extracellular vesicles and a convenient tool to perform proteomic analysis in a quick and reliable manner. FunRich is an alternative to the currently available software that allows the generation of graphical representations (Venn diagrams, pie charts, bar graphs, or heatmaps) as well as protein–protein interaction network visualization. Vesiclepedia (Ver. 3.1; 09 January 2015) is a manually curated compendium of molecular data (lipid, RNA and protein) identified in different classes of EVs. Currently, Vesiclepedia comprises 92 897 proteins, 27 642 mRNAs, 4934 miRNAs, and 584 lipid entries encompassed from 538 independent studies that were published over the past several years [137]. The tool exRNA is a research portal aimed at discovering fundamental biological principles about the mechanisms of exRNA generation, secretion, and transport, and to investigate the potential for using exRNAs in the clinic as therapeutic molecules or biomarkers of disease [138].

## 8. An Integrated Collection of Small RNA Research Tools

Given the wide range interest in small RNAs, bioinformatics tools to assist researchers dealing with small RNA research are undoubtedly needed. After a first wet-lab step consisting in the evaluation of small RNA expression by means of NGS approaches, downstream data analysis might be performed using different tools: small RNA toolkit [139], miRDeep [140] and miRDeep2 [141], miRanalyzer [142,143], SeqBuster [144], DARIO [145] UEA sRNA workbench [146] and ShortStack [147]. In certain cases, some of these tools include other prediction tools for novel microRNAs or isoforms of miRNAs (isomiRs) quantification. However, they do not generally offer functional downstream analysis or data visualization. Some exceptions are mirTools 2.0 [148], which includes a target prediction and a functional analysis module, UEA sRNA workbench which includes a visualization tool and sRNAbench for multi-species analysis [149]. sRNAtoolbox is a set of independent but interconnected tools for expression profiling from high-throughput sequencing data, consensus differential expression, target gene prediction, visual exploration in a genome context as a function of read length, gene list analysis, and blast searches of unmapped reads.

Briefly, sRNAtoolbox contains the following tools:

*sRNAbench*: expression profiling of small RNAs, prediction of novel microRNAs, analysis of isomiRs, genome mapping and read length statistics. Samples are analyzed individually.

*sRNAde*: detection of differentially expressed small RNAs based on three commonly used programs: DESeq [150], edgeR [151] and NOISeq [152].

*sRNAblast*: aimed at determining the origin of unmapped or unassigned reads by means of a blast search against several remote databases. The results can either point towards contamination sources or biologically meaningful information, such as the presence of unexpected viral or bacterial RNA molecules.

*miRNAconsTarget*: consensus target prediction on user-provided input data based on Miranda [153], PITA [154] and TargetSpy [155] for animal microRNAs and PsRobot [156] and TAPIR (target prediction for plant microRNAs) [157] for plant microRNAs.

*sRNAjBrowser*: visualization of sRNA expression data in a genome context using jBrowse [158].

*sRNAjBrowserDE*: visualization of differential expressions as a function of read length in a genome context using jBrowse.

*sRNAfuncTerms*: enrichment of functional annotations in animal and plant target genes not only for a set of microRNAs but also for all its combinations (microRNA modules).

*sRNAfuncTargets* (workflow): cross-species microRNA target prediction and enrichment analysis of functional annotations.

All tools can be used independently or for the exploration and downstream analysis of sRNAbench results. The web-interface interconnecting all these tools is available at http://bioinfo5.ugr.es/srnatoolbox [159].

## 9. Conclusions

The discovery that miRNAs circulate in body fluids has generated a great interest in their potential use as disease biomarkers. Circulating miRNAs are not cell-associated and generally escape from degradation by endogenous ribonucleases owing to their localization within membrane-structured bodies as well as protein and lipid complexes. The functional roles that these circulating miRNAs might play in the human body even at distant sites have been and will continue to be the focus of future studies. In fact, several highly abundant miRNAs can be present at the same time in multiple fluids, whereas some miRNAs are enriched only in specific fluids. Extracellular miRNAs can potentially interact with recipient cells via a number of different processes, including direct fusion, passive uptake, and-receptor mediated interactions [160]. Over the last several years, the presence of circulating miRNAs has been associated with a variety of medical conditions and linked to many pediatric diseases such as the ones discussed in this review. The expression level of circulating miRNAs can be easily assessed by various methods, mainly quantitative polymerase chain reaction, which allows signal amplification, and next-generation sequencing.

Finding informative biomarkers from biological fluids will be not only key to the understanding of the physio-pathological processes of diseases, but will be also the next crucial challenge for the development of new therapeutic strategies.

## Figures and Tables

**Table 1:** List of analysis tools and databases for circulating extracellular miRNAs, web links and year of publication. Resources listed in the table have been described in the review.

Bioinformatics Resources	Year	Web Link	Reference
miRandola	2012	http://atlas.dmi.unict.it/mirandola/index.html	[56]
miRò	2009	http://ferrolab.dmi.unict.it/miro	[133]
Evpedia	2012	http://evpedia.info	[134]
ExoCarta	2009	http://www.exocarta.org	[135]
FunRich	2015	http://www.funrich.org	[136]
Vesiclepedia	2011	http://www.microvesicles.org	[137]
exRNA Research Portal	2014	http://exrna.org	[138]

## Abbreviations

ALT	Alanine aminotransferase
ASCA	anti-Saccharomyces cerevisiae antibodies
ASD	Autism spectrum disorder
AUC	Area under the curve
BA	Biliary atresia
CD	Crohn’s Disease
CFLD	Cystic fibrosis-associated Liver Disease
CHB	Chronic hepatitis B
CRP	C-reactive protein
CVD	Cardiovascular diseases
FMD	Flow-mediated dilatation
HBeAg	Hepatitis Be antigen
HBV	Hepatitis B virus
HC	Hypercholesterolemia
HCC	Hepatocellular carcinoma
HDL-C	High density lipoprotein-cholesterol
IBD	Inflammatory Bowel Disease
IgA	Immunoglobulin A
IgAN	IgA nephropathy
IgG	Immunoglobulin G
INRG	International Neuroblastoma Risk Group
isomiRs	isoforms of miRNAs
LDL-C	Low density lipoprotein
miRNAs	microRNAs
NGS	Next generation sequencing
NMP1	Nucleolar phosphoprotein
NO	Nitric oxide
NS	Nephrotic syndrome
OGTT	Oral glucose tolerance test
OSA	Obstructive sleep apnea
qPCR	Quantitative Polymerase Chain Reaction
ROC	Receiver Operating Characteristic
ssRNA	single-stranded RNA
T1D	Type 1 diabetes
TAPIR	target prediction for plant microRNAs
TC	Total cholesterol
TLRs	Toll-like Recetors
UC	Ulcerative Colitis
WHO	World Health Organization
